# Persons with disabilities as experts-by experience: using personal narratives to affect community attitudes in Kilifi, Kenya

**DOI:** 10.1186/s12914-018-0158-2

**Published:** 2018-05-08

**Authors:** Joseph K. Gona, Charles R. Newton, Sally Hartley, Karen Bunning

**Affiliations:** 1 Centre for Geographic Medicine Research (Coast), Kenya Medical Research Institute, P.O. Box 230-80108, Kilifi, Kenya; 2 Department of Psychiatry, Oxford University, Oxford, UK; 3 Sydney University, Sydney, Australia; 4 University of East Anglia, Norwich, UK

**Keywords:** Disability awareness, Community, Attitudes, Experts-by-experience

## Abstract

**Background:**

The last decade has seen improved public awareness of disability in sub-Saharan Africa. However, negative and stereotypical views of disability still persist in many communities. We conducted a study to promote awareness of disability in rural Kenya, using a process of reflection and education. This paper reports on the second aspect – education. The research question was: How can personal narratives of living with disability affect community attitudes and responses to disability?

**Methods:**

A qualitative phenomenological approach was adopted. Twenty community-based groups involving 249 participants took part. Each group participated in one focus group discussion at baseline, to explore the members’ personal experiences and views of disability. The intervention involved three adults with disabilities sharing their personal narratives with each group. After the intervention, repeat focus group discussions were conducted with each group. Thematic analysis was carried out according to the framework method.

**Results:**

The emergent framework consisted of four main themes, organised as opposing constructs: ‘burden’ and ‘agency’, ‘sub-human’ and ‘human’. ‘Burden’ focused on the perceived hopelessness of the situation. Post-intervention revealed greater support for the ‘agency’ of persons with disabilities, evidenced by what the person could do, rather than their inability, and the relevance of support. The ‘sub-human’ to ‘human’ construct captured dehumanising and discriminating practice towards persons with disabilities on one side, and recognition of the person and inclusion in the community on the other. Whilst support and empathy were evident at the pre-intervention stage, post-intervention revealed greater recognition of people with disabilities as fellow human beings.

**Conclusion:**

This study provides a proof of concept regarding the deployment of persons with disabilities as agents for change. Exposure to experts-by-experience provided community groups with opportunities to reflect on, examine and adjust their views on disability in this rural part of Kenya. The sharing of personal narratives appeared to resonate with group members, to encourage recognition of the person and not just the disability, and to move their resolve toward ideas for collective action. Further research is needed to assess the effects of such interventions.

**Electronic supplementary material:**

The online version of this article (10.1186/s12914-018-0158-2) contains supplementary material, which is available to authorized users.

## Background

How a community views, understands and responds to disability is important to the everyday life of people who have disabilities. Cognitive maturity [1–3], cultural beliefs and practices [4], information availability [5, 6] and exposure to people who have disabilities [7] are influential factors [8] . Childhood representations of disability tend to be negative and typically associated with an undesirable condition [9], resistant to parental influence and closely aligned to an individual or medical model, with attention given particularly to physical and biological factors [9, 10]. Federici et al. proposed that this was evidence of a ‘cognitive mechanism’ underlying the conceptualisation of disability [2]. As cognitive maturation occurs, there is engagement with alternative perspectives on disability [1–3]. However, it was observed that parents resort to early mental representations in response to open-ended questions, as opposed to closed question responses that were aligned to the social model [1] .

Cultural learning is also important. Traditional beliefs have tended to associate disability with negative images and experiences [4], although some variations have been reported in East Africa, e.g., people with physical disabilities were perceived as pacifiers of evil spirits [4], while autistic spectrum conditions (ASCs) were attributed to evil spirits, witchcraft and curses [11]. Some of the distaste associated with disability in sub-Saharan Africa may lie in the cultural explanations that imply: breach of social conventions, e.g. in Botswana [12], Ghana [13], Kenya [14–16], Tanzania [17], Malawi [18]; external, preternatural forces, e.g. in Kenya [14–16], Namibia [17],; and the will of God [19–22]. Negative and sometimes fearful images associated with disability, not only hinder acceptance of the person who has a disability, but also undermine possibilities and opportunities for the person [23]. Corrigan [24] and Corrigan & Watson [25] asserted the cognitive, affective and behavioural components of stigma, whereby people who share a particular characteristic that marks them out as different, e.g. an inability to walk, are cognitively appraised by others, leading to a possible negative emotional response and discrimination [26].

The question of how such stigma spreads across a community remains. Dipple et al. considered the relevance of ‘stigmergy’, a term originating from the French language, to the human world [27]. Thus stigmergy provides an explanation for the tendencies that exist within a community in their responses to persons with disabilities and their close family members. At its most extreme point, community responses to people with disabilities may descend into abuse, neglect and exploitation. For example, discriminatory practices reported in Nigeria [28] and Uganda [29] included physical, sexual and emotional violence, human trafficking, ritual killing and alms begging.

As a counter point, purposeful encounters with people who have disabilities appear to be critical to the formation of positive attitudes. Allport’s early ‘contact hypothesis’ proposed that increased interactions by one social group with another would promote more positive attitudes and decrease prejudice [30]. This has been borne out in studies of children’s attitudes towards disability [31, 32]. A systematic review of 35 studies revealed that children’s attitudes were positively associated with the social contact experienced with people who have disabilities [33]. This was corroborated in a more recent cross-sectional survey of over 1800 children, in which empathy for and underlying anxiety about interacting with people with disabilities were reported mediators of the contact-attitude link [34].

Disability awareness training often involves interacting with people who have disabilities, combined with activities that have a more cognitive focus, e.g. engaging with new information and participating in discussions that challenge stereotypes. By increasing exposure to the people who are the very objects of devaluation in a positive and informed way, previous evaluations may be challenged [35–37]. Krahè & Altwasser [38] found that combining cognitive and behavioural activities, (where students took part in sporting activities led by Paralympic athletes), improved attitudes toward persons with physical disabilities. In direct contrast, no change was associated with the cognitive condition only and the control condition. Similarly, Papaioannou et al., used disability simulation in sports activities, some lectures and video presentations, to bring about positive change in attitudes [37]. A systematic review of interventions targeted at schools in Canada revealed shifts in attitudes and behaviours of the children associated with five basic approaches to disability awareness: (i) social contact; (ii) simulation; (iii) curriculum; (iv) multi-media curriculum; and (v) multiple components [39]. Documentary evidence of living with disability and discursive interactions were found to improve disability awareness in teenage boys in Australia [37]. In an epilepsy education programme conducted on the Kenyan coast to reduce the treatment gap, traditional songs with positive depictions of epilepsy were used to change community perceptions and to promote new understanding [40].

Disability awareness training features in the ‘empowerment’ component of the Community-Based Rehabilitation (CBR)/Inclusive Development guidelines, which cuts across the other four components of health, education, livelihood and social [41]. However, there is a dearth of evidence for CBR initiatives generally, with published studies being criticised for a lack of research rigour, and for being overly descriptive or theory-based [42]. In their recent Systematic Review, Lemmi et al. [43] focused on assessed impact. Modest benefits were suggested for people with mental disabilities and their caregivers, although the authors acknowledge ‘methodological constraints’ (p.6) in the studies. More recently, consultations on how best to address the evaluation of CBR have favoured a flexible and iterative approach [44].

Based on the evidence that personal encounters with people living with disability have positive influence, and in harmony with the recommendations of the CBR guidelines, a project was set up to engage neighbourhood communities in a process of reflection and education. The first aspect - reflection, focused on community beliefs about disability causation and the attendant challenges [14]. The current paper reports on the second aspect – education. The aim was to develop and evaluate the effects of a disability awareness intervention on the values, perceptions and understanding of community groups in a rural part of Kenya. The research question was: How do personal narratives of ‘experts by experience’, i.e. persons with disabilities, affect the perceptions, values and understanding of community groups?

## Methods

With reference to Creswell et al.’s description, a phenomenological approach was adopted [45]. Thematic analysis of focus group discussions data was conducted before and after a disability awareness intervention.

The setting was Kilifi County, one of the poorest areas in Kenya, with a poverty level of 71% [46]. Situated on the Indian Ocean coast it covers a large rural area where subsistence farming dominates. Characteristics of the region include low levels of nutrition, inadequate control of infectious diseases, poor enrolment in schools, limited literacy amongst adults generally and unreliable rainfall. Typical dwellings are of mud construction with a thatched or iron sheet roof, no power supply or running water. Per capita, the average income for a household (typically parents plus six children) is Ksh1, 000 per month – less than $13 USD [46]. Languages spoken include Mijikenda and Swahili. Christianity is observed by about 70% of the people, traditional religious practices by 20% and Islam by about 10%. Based on a county-wide population of 1,109,735 and using a 15% prevalence of disability [46], it is estimated there were 166,460 people with disabilities in Kilifi County.

### Sample

The study was conducted across five constituencies of Kilifi County, Kenya. They included Magarini, Malindi, Kilifi North, Kilifi South and Ganze. The focus was on two types of established community groups, namely community health worker groups (CHW), who are volunteers brought together to support the work of health dispensaries and health centres [47], and women’s groups (WG), who are registered with the Kenya Women Finance Trust (KWFT) [48]. The two types of community groups were targeted for their convenience, being readily accessed, formally constituted and meeting at regular intervals, for their ongoing community engagement, and for their stable membership. The latter point was considered critical to the facilitation of focus group discussions. Inclusion criteria used in the study required that the group should:

- Be formally constituted as a CHW or WG;
- Be active, having regular meetings, at least once a month;
- Have a consistent membership as evidenced by attendance of meetings;
- Have contact with people with disabilities.

**Fig. 1:**
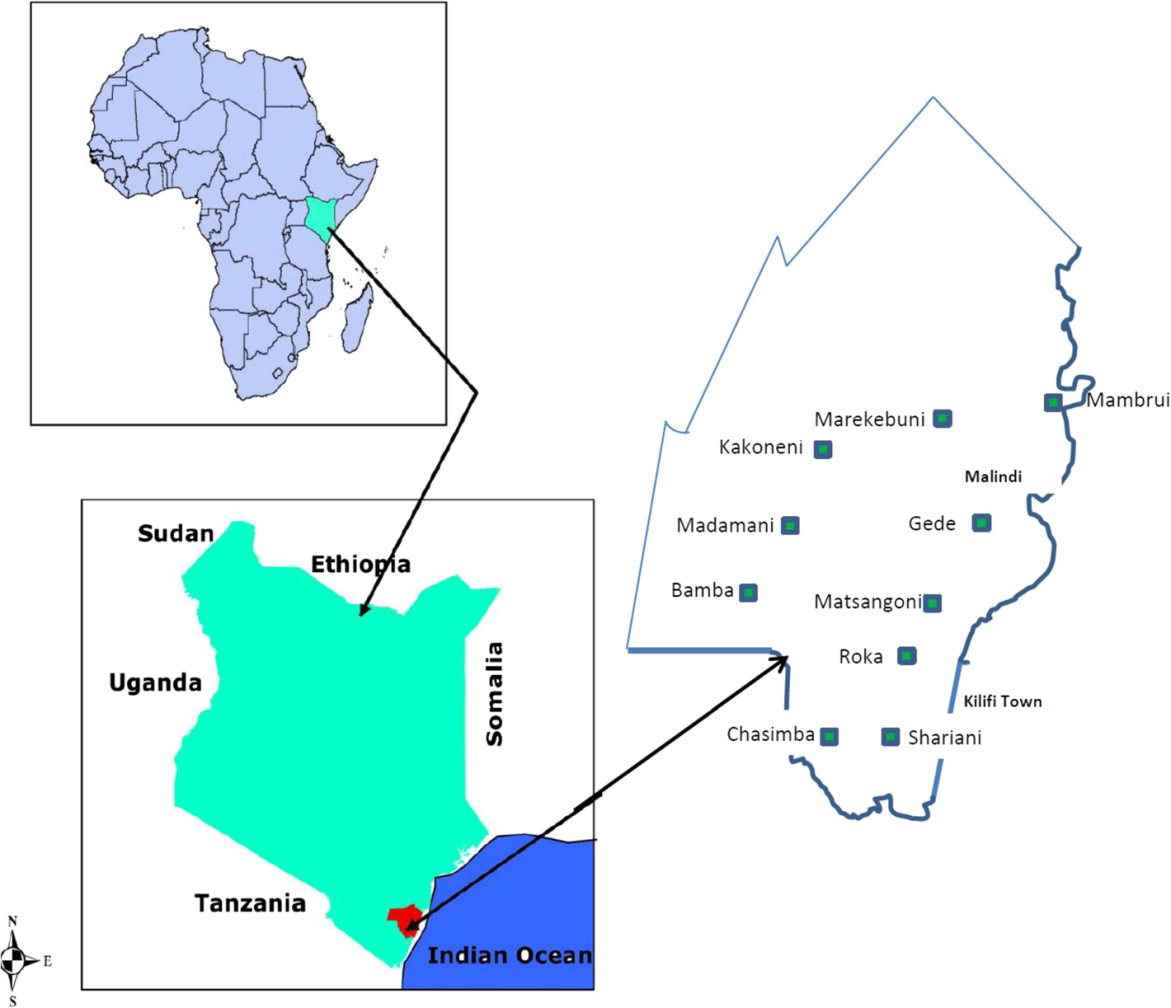
Geographical map og kilifi County showing the sub-locstions across the five constituencies

Recruitment of the groups was facilitated by Pambazuko Disability Initiative, a community-based organization involved in providing support for children with disabilities in Takaungu, a sub-location in Kilifi County. Building on their existing links with WGs and CHWs, they identified 20 groups (10 CHW and 10 WG) who met the criteria in ten sub-locations. Each community group had around 15 members making an estimated total of 300 participants. The representative from Pambazuko approached each community group and arranged for the Project Researcher to attend a meeting for purposes of providing information about the project and soliciting their consent to participate. Each member gave their consent separately, recorded by signature, personal mark of thumb print on a consent form designed for the purpose. Fig. 1 shows the sub-locations of the groups.

### The intervention

The intervention aimed to promote disability awareness in the community. Based on Allport’s contact hypothesis [31], the rationale for the intervention was concerned with exposing members of the community groups to people living with disabilities. Disabled Persons and Parent Organisations are not common in this rural part of Kenya and therefore it was decided to recruit adults with disabilities via local networks A retired special educational needs teacher, familiar with local organisations for people with disabilities, and conversant in the local languages, as well as Kenyan Sign Language, recruited five adults living with disabilities, termed ‘experts-by-experience’, to the group. Individuals were identified via special schools and units and invited to take part in the development and delivery of a disability awareness training initiative. The group was comprised of:

- V, male, aged 45 years with cerebral palsy;
- W, female, aged 36 years with hearing impairment;
- X, male, aged 20 years with intellectual disability and traits of autism spectrum condition (ASC);
- Y, female, aged 20 years with hearing impairment;
- Z, male, aged 25 years with intellectual disability

The group met for three hours every day over a six-week period to develop their personal narratives of living with a disability for sharing with the community groups. The first author (JKG) facilitated the training in partnership with the special needs education teacher. JKG organised for the training facility, and made sure the expert group reached the training venue on time. The special needs teacher rehearsed with the group and did sign language translation.

1. Each member of the expert group was first given an opportunity to narrate his or her life story.
2. Then JKG and the special needs teacher assisted in putting their stories in organised sequences.
3. Each member was given a topic to talk about to minimise repetition: V was to talk about social life experiences, W about challenges of doing business, being independent, X to talk about challenges of being in school and living in a homestead. A homestead in this context means a structured cluster of houses belonging to relatives of the same grandparents.

After the six weeks, two members of the expert group, Y and Z, opted to pull out of the study. Y did not have basic knowledge of Kenyan Sign Language and Z relocated to another place with his parents. The special needs teacher interpreted the sign language to members of the community groups who participated in this study.

The experts-by-experience were invited to share their stories with the community groups. Meetings started with prayers. Then the persons with disabilities proceeded to relate their stories one at a time. V was first to give his story about when he was in school, after school and his struggle in trying to get a wife. W was next in giving her story. She had a hearing impairment so the special needs teacher translated her sign language to the participants in the community groups. She based her presentation on personal experiences of starting a business within a community and being discriminated against within a family setup. X talked about experiences in school, and the challenges experienced at home. After the narratives, the community group members were given the opportunity to discuss issues and pose questions to the experts-by experience. The expert group was given financial compensation for their time, both for the training and the visits to the community groups during the intervention period.

### Data collection and management

Focus group discussions (FGD) were conducted with each of the twenty community groups, pre-and post-intervention. The meetings were arranged by the Pambazuko representative in communication with group’s chairperson. The meetings took place in usual meeting place for each group, and refreshments were provided. The Chairperson opened the meeting and led the group in a prayer. The discussion was then introduced by the first author (JKG) who followed an interview schedule consisting of topics and a questioning route. At baseline, the groups were also asked to reflect on the causes and challenges of: (a) having a disability; and (b) caregiving for someone who has a disability. This is reported in a separate paper [14]. They were encouraged to talk about their experiences of children and adults with disabilities in their own communities and to consider the role of the community in the lives of such individuals.

Post-intervention visits were conducted approximately 2 months after the group had been exposed to the personal narratives of the experts. The groups were asked to consider the narratives shared by the experts by experience and what they had learned. The schedule of questions is provided in Additional file 1.

Table 1 shows the attendance of the focus group discussions for the different group types – CHWs and WGs. The WGs had a higher membership attendance rate (pre: *n* = 134; post = 145) compared to that of the CHW (pre = 114; post = 118) with a higher median attendance (CHW = 11; WG = 14).

**Table 1:** Summary of community group attendance: pre- and post-intervention

Group-type	No. of groups	Pre-intervention	Post-intervention
		Range (median)	Range (median)
CHW	10	8–15 (11)	8–20 (11)
WG	10	11–17 (14)	12–18 (14)

The WG were comprised of all women and the CHWs had female to male ratio of 6:9. Discussions were carried out in the preferred language of the group and recorded on a digital audio device. With reference to the questioning route, questions were posed to the whole group initially to invite spontaneous responses. In order to establish a fair distribution of turns, this was followed by encouraging others to add to or comment on the views previously expressed. The duration of the FGDs was about two hours.

The recorded data were uploaded to a computer and transcribed in the language used by the group, before translation into English; imported to the data management programme NVivo 10 for storage and analysis. To ensure accurate representation of meanings in the translated copies, checks were carried out during the analysis period. This involved the first author (native Kenyan and speaker of the local languages) and the last author (English-speaking, United Kingdom (UK)-based visitor to Kenya) reviewing the transcripts, querying emergent concepts and their meanings, and using back translation as appropriate.

### Data analysis

‘Framework’ was the method of analysis used to evaluate impacts associated with the intervention [49]. The entire data set was analysed following a structured process of: familiarisation with the transcripts initially, through repeated readings; identification of a thematic framework by reviewing the data, by noting key concepts and establishing a first generation of nodes using NVivo-10; ‘indexing’ whereby the data were explored and assigned to nodes and sub-nodes as appropriate; application of the ‘index’ whereby transcript excerpts were assigned to the different node levels; and finally, mapping the range and nature of phenomena, creating typologies and finding associations between them, and interpreting the data as a whole. The first and last authors analysed the data separately, coming together on several occasions during the process to compare their separate analyses. The first author, a native of this area of Kenya, applied his local knowledge and experience to the analysis, while the last author, a UK-based, regular visitor to Kenya, provided a more remote and impartial stance from which to analyse the data. Differences in frameworks were discussed, nodes and their relationships were reviewed, until agreement was achieved.

## Results

**Fig. 2:**
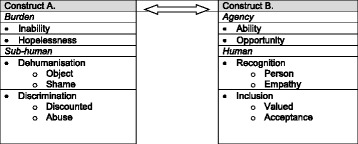
Schematic representation of organizing and basic themes as opposing constructs

The emergent framework consisted of four organising themes: Burden; Agency; Sub-human; Human. They were arranged as pairs of opposing constructs along a continuum (A. to B.), i.e. burden vs agency; sub-human vs human as shown in Fig. 2 which captured the possibility of movement between them. Subordinate constructs were identified at lower levels of each main construct pair, as indicated by filled bullet points. Only the sub-human vs human construct was further sub-divided into opposing pairs of basic themes, as indicated by unfilled bullet points.

With reference to the framework in Fig. 2, the results are presented in a series of tables. Each table focuses on a different pair of subordinate constructs, labelled in the side margins of the table, from within an organising pair of constructs (labelled at the top of the table). Basic themes are shown in Tables 3 and 4 only (Sub-human vs Human) indicated by unfilled bullet points within a column, where data from pre- and post-intervention are presented. Immediately after each quote, the source is given in square brackets, i.e. the group type and participant number, and the pre- or post-intervention status indicated by − 1 or − 2 respectively.

### ‘Burden vs agency’ construct

The ‘burden vs agency’ construct was composed of two pairs of opposing themes, referred to as sub-constructs:

- *Inability* ***-*** *ability;*
- *Hopelessness-opportunity.*

‘Burden’ was defined as a problem that affected other people around the individual with a disability, with particular mention of the mothers as the primary caregivers. In the context of limited resources, both economic and psychological, there was the expressed view that having a person with a disability in the family, and in the wider community, was a source of difficulty. Reference was made to negative images of disability and disturbance to the well-being of others, both caregivers and members of the community. It was the case that disability was associated with ‘burden’ defined by ‘hopelessness’ and ‘inability’, almost exclusively at the pre-intervention stage, with 81 references made pre-intervention and only 3 references post-intervention. However, there was also recognition of the ‘agency’ of people with disabilities, defined as the capacity of an individual or group of individuals to act in the world. At post-intervention, ‘agency’, defined by ‘ability’ and ‘opportunities, emerged more emphatically with 193 references pre-intervention and 230 references post-intervention.

#### Inability-ability

On one side of this sub-construct was *inability*, which captured a lack of capacity or what the person could not do, and what was perceived to be missing in the person. This was associated with dependence on others for care and support needs. The other side of the construct was *ability*, which referred to the individual having skills. It focused on the things the person could do, e.g. walking, learning, drawing water, earning money.

‘Ability’ was recognised both pre- and post-intervention. Pre-intervention discussions tended to focus on persons with disabilities who were known to the respondents. For example, references were made to individuals who used manual signing for communication, whose work involved digging the land and carrying water. Post-intervention the discussions focused more on the abilities of the experts who had presented their personal narratives. However, the opposing construct of ‘inability’ was expressed almost exclusively in the pre-intervention groups, with the exception of just 1 reference made post-intervention:“ …having limitations and there are things they cannot do.” [CHW10–2]. However, the use of ‘things’ implies a more measured response that is different from the more exacting responses in the pre-intervention discussions. Here the emphasis was on what the person could not do – their lack of capacity, as exemplified by the use of intensifiers as in “*totally* disabled”, and qualifiers as in “…cannot *even* eat”. Negative structures, such as “not”, were used in the pre-intervention references to people with disabilities. There was a shared perception of them being incomplete. Post-intervention, disability and ability were not mutually exclusive. That is, the respondents cited examples from the experts-by-experience, concluding that disability was not inability. Table 2 provides supporting quotes from the transcripts.

**Table 2:** Burden vs Agency construct: Inability-Ability

Burden		Agency	
Inability	‘She is totally disabled…. he is the kind of a disabled person who is paralyzed, she spills saliva.’ [CHW1–1] ‘if the child is not moved to another place then he will remain at the same position.’ [CHW2–1] ‘the child is not able to do anything. The child cannot even eat.’ (CHW4–1] ‘you call him and he just looks at you. … he can’t say any word. He just hmmms ‘mmm mmmm’ that how he talks.’ [WG2–1] ‘… he is not normal you can put him in the same category with the mad people.’ [CHW2–1] ‘a disabled person is one who is not complete others lack parts of the body due to stroke’ [CHW6–1]	‘… he is blind and when he goes to the garden he can dig and also fetch water with the other children.’ [CHW10–1] “When he is hungry he will give you a sign. He will wait until you are looking at him then he will show (gestures eating with his hand), then you will know that he wants to eat” [CHW3–1] ‘F- said he went to school to class eight, he trained in a profession, and he was employed.’ [CHW5–2] ‘…regardless of the disability, people with disabilities can learn in school.’ [CHW6–2] ‘They have abilities, though not as the others but they have abilities.’ [CHW11–2] ‘They know how to work. When they ask for a job, don’t look at his disability. Look at his abilities; look at what he can do.’ [WG7–2]	Ability

1 = pre-intervention; − 2 = post-intervention

#### Hopelessness**-**opportunity

*Hopelessness* captured the sense of giving up and the desire to have the individual with a disability gone. Associated with this was a lack of ideas or momentum to act in a different way. The antithesis of this was *opportunity,* which encapsulated the idea of supporting the person with a disability to take part in activities, e.g. attend school, regardless of their particular challenges. This implied putting help in place, and helping to circumvent some of the observed difficulties.

Pre-intervention, the focus group discussions told of situations that implied *hopelessness*. A sense of despondency was conveyed in the stories shared, and even of individuals with disabilities being rejected. There was some notion of supporting the child with a disability to be “in the world” at the pre-intervention stage, but this was augmented considerably in the post-intervention discussions. *Opportunity* was defined as support and calls to action by the community. Greater awareness of opportunities was shown post-intervention, with consideration of health, education and employment, which underpinned the groups’ growing sense of the ‘agency’ of people with disabilities. Calls to action included having a community meeting as “…one voice...” and getting others to listen to the stories of people with disabilities. Table 3 provides supporting quotes.

**Table 3:** Burden vs Agency construct: Hopelessness-Opportunity

Burden		Agency	
Hopelessness	‘…she was thrown away. …’ [CHW2–1] ‘…she would put her near the fire so that she can die faster because she was tired of the work.’ [CHW1–1] ‘the parent usually has lost all hope regarding the child.’ [CHW7–1] ‘… without that (sponsors) they are just left alone like how we see them.’ [CHW7–1] ‘So what can we do, we are not able to help him. People don’t like him.’ [WG1–1] ‘… the child had jiggers (parasitic infestation of fleas) on her hands … she had to move from one place to the other using her bottom. She stayed with the jiggers in her hands without being treated and she eventually died because of that.’ [CHW2–1]	‘We look for people to help the child so that she can feel that she is in the world.’ [WG4–1] ‘He will say what he wants and that he feels he needs a wife. So a wife should be found for him to marry. ‘You will have done justice to him’ [CHW7–2] ‘… we have to be close to them so that we can understand them so that we can address their needs.’ [CHW1–2] ‘…educate them so that they can have education. …. they can cater for their future lives.’ [WG7–2] ‘If I have some work, like that young man who kept poultry, I can call him and give him the job.’ [WG3–2] ‘We have to know about their health, their wellbeing… If he is sick……. if he can be carried should be taken to a doctor.’ [CHW1–2] ‘…. a person with a disability. We could take him to the meeting and he explains what he goes through in his life…they will see and hear what he has gone through.’ [WG5–2]	Opportunity

### ‘Sub-human vs human’ construct

The theme of ‘Sub-human’ denotes a focus on the disability before the person, which dominated group discussions before the intervention (references pre-intervention: 129; post-intervention: 13).. In contrast, the theme ‘Human’ refers to recognizing the person before the disability. This included attributing positive value to the individual and viewing him/her as a member of the community (references pre-intervention: 56; post-intervention: 222). The ‘sub-human’ vs ‘human’ construct was composed of two pairs of sub-constructs:

- *Dehumanisation* ***-*** *recognition;*
- *Discrimination-inclusion.*

#### Dehumanisation-recognition

D*ehumanisation* included references to people with disabilities that rendered them as negatively valued. The words used for people with disabilities referred to the type of disability as a collective noun, with neglect of the person. Practices were described that were devoid of humanitarian effort, e.g. chained up, tied, food passed with stick. The individual was rendered as an object, without human feelings or potential. The antithesis of this was *recognition*, which acknowledged the person in his or her own right. There was notice of the person’s struggles and difficulties, with expressions of empathy for their experiences.

The terms used in the groups before the intervention, depicted persons with disabilities as ‘things’ belonging to the ki-vi class of nouns in the Kiswahili language. Individuals were referred to as “kiwete” (crippled), “kiziwi” (deaf) and “bubu” (dumb), or “akili punguani” (mental retarded). This had the effect of classifying the person with a disability as *sub-human*, viewed as an ‘object’ or “grown like that of an animal”. However, there was also ‘recognition’ of the ‘person’ as stories of familiar individuals were told, e.g. “…raise my hand to greet him…”, ‘“the first person to get the news”. Post-intervention, the terms used previously in relation to people with disabilities were revised towards greater ‘recognition’ of the person, before the disability, e.g. “we are all human beings…” Realisation of the sameness of persons with and without disabilities was expressed in relation to their religious convictions, e.g. “created by the same God” also.

The basic theme ‘shame’ referred to the indignities suffered by people with disabilities and some of terrible treatment meted out by others. The stories were told both at pre-and post-intervention; however, greater ‘empathy’ characterised the stories at post-intervention, although not exclusively, e.g. “…you will sympathise with him…” said one respondent describing someone in her local community before the intervention. However, after hearing the narratives of the experts-by-experience, the group members expressed affect as well as awareness of the person’s emotions, e.g. “…saddened by their stories…” and “…he also has emotional feelings”. Table 4 gives supporting quotes.

**Table 4:** Sub-human vs Human construct: Dehumanisation-Recognition

Sub-human		Human	
Dehumanisation	○ Object	○ Person	Recognition
	‘I have seen one and he was “kiwete” (crippled) and had to use a wheelchair to move from place to place.’ [WG5–1] ‘There are the “bubu” (dumb) and “kiziwi” (deaf).’ [CHW2–1] ‘There is a boy who has been nicknamed “Kahindi Kadzitswa” (Kahindi with a small head).’ [CHW9–1] ‘You find that his body has grown like that of an animal.’ [CHW10–1]	‘when I pass by there I raise my hand to greet him and he responds by doing that also.’ [WG6–1] ‘he feels he is just like the other normal people, he is not neglected and he is the first person to get the news.’ [CHW10–1] ‘…it is important that the community should know that persons with disabilities are not ‘viwete’ (crippled), they are people who lack something in their body formation.’ [CHW3–2] ‘We should know that we are all human beings created by the same God. Whoever gave us all the limbs is the one who also denied them the limbs.’ [WG7–2]	
	○ Shame	○ Empathy	
	‘…locks the child up in a house and when she is being given food she pushes the food to her using a stick….’ [CHW2–1] ‘There is a boy down here who is tied using chains on his feet.’ [CHW2–1] ‘… she is left at the bed the whole day and the whole night. She is just fed and be left there.’ [WG9–1]	‘I learned that there is need to have good regard for them like any other person. If there is need you teach him something, teach him. We can also learn from them; they can teach us that even this one I can do. [WG4–2] ‘They know how to work. When they ask for a job, don’t look at their disability. Look at his abilities; look at what he can do. .’ [WG1–2]. ‘I was close to him and he held my hand and I felt that he was strong. So I learned that he also has emotional feelings.’ [CHW5–2]	

#### Discrimination-inclusion

*Discrimination* described the ways in which the person was treated differently to their peers, being ‘discounted’ or ‘abused’. Opportunities were constrained and exclusions were commonplace, rendering the person disempowered and disenfranchised. The opposite of discrimination was ‘inclusion’, which captured being ‘valued’ and establishing ‘acceptance’ in everyday life.

The discussions pre-intervention revealed tales of ‘discounted’ individuals who were acknowledged to be “…valueless in the family” and discriminated against when earning a living, e.g. “…the (fried) fish will not be bought…” Other stories told of ‘abuse’ which included exploitation also. Post-intervention there was deliberate consideration of the actions needed to counter the ‘discounted’ ways of responding to persons with disabilities. Actions that valued the individual included “...showing them love…”, “…care(ing) for them…”, and “…know(ing) about their health, their wellbeing…” – all expressions of human need. Finally, there was ‘recognition’ of a need for ‘acceptance’ such that persons with disabilities should be treated in a similar way to those without disabilities, with a call to “…sensitise the community…” Table 5 gives details.

**Table 5:** Sub-human vs Human construct: Discrimination√Inclusion

Sub-human		Human	
Discrimination	○ Discounted	○ Valued	Inclusion
	‘They view the child as something that was not lucky to be born that they did not have any need for it. The child is valueless in the family.’ [CHW3–1] ‘So she can fry the fish but at the end of the day the fish will not be bought.’ [WG4–1] ‘He did pass through a lot of problems because when a visitor arrived at their home he was not allowed to come to the sitting room.’ [CHW6–1]	‘....we are supposed to love them,’ [WG7–2] ‘….maybe we buy them T-shirts for each and let them wear them. This is showing them love. At least they will see; that is to start with.’ [CHW5–2] ‘… these people do not deserve to be discriminated. They need to be loved; care for them like others….’ [CHW10–2] ‘Eating together with the disabled… one parent might say, ‘Look! My child is eating with those people; he is not as I was thinking about.’ [CHW3–2]	
	○ Abuse	○ Acceptance	
	‘She was raped and then later on she was taken to the hospital..’ [CHW2–1] ‘They use the child in a bad way because he is the one who plays the drum when there are drunkards at their place...’ [CHW6–1] ‘… other people are not welcoming in their houses …. chasing him away or even beating him up.’ [CHW11–1] ‘When he went out to play with the other children he was beaten up a lot …….’ [CHW6–1]	‘We should sensitize the community that what we do to the non-disabled, we should do the same to the disabled.’ [WG1–2] ‘the community should be made aware that the disabled are part and parcel of the community. So the most important thing is to accept them and that he was born with that condition and that he is like any other child in the family.’ [CHW4–2]	

## Discussion

Occurring mainly in pre-intervention group discussions, dominance of negative images and the use of language that tended to focus on the physical or visible characteristics of disability were suggestive of the individual or medical model [9]. The open-ended questions asked of the groups may have triggered access to early mental representations of disability that were formed in childhood as suggested by Federici, Meloni and colleagues [1, 2]. This would imply the presence of an underlying cognitive process at work [2]. In seeking to formulate a clear and consistent response to the disability focused questions, the respondents resorted to the most immediate explanation available in their mental lexicon [1]. The models act as reference points, which inform the development of social responses and relations [1]. In the absence of a more social representation of disability, the respondents have attributed disability to deviation from more typical biomedical structures and functions [9, 10].

Cultural beliefs and traditional practices have been commonly associated with negative images [12, 15–18] and concepts that inspire fear in others [4]. Thus the explanations of disability that were espoused by members of this East African community, e.g. preternatural forces [12, 15–19], may have shaped the images of abnormality and dysfunction that featured in the groups’ discussions. As suggested by Meloni et al. [1], the polarisation of the individual/medical and social models is more a product of culture than cognition, which infers a learning process. Inadequate coverage of information about disability across rural communities [5, 6] means that alternative models or ways of understanding disability may not have been available. Thus, it could be said that the cultural construction of disability upheld the idea of the individual model, at least in part, by viewing disability as a direct consequence of a supernatural event [15–18]. In contrast, breach of social conventions [14, 17] placed responsibility on the actions of other people, and therefore deflected the focus from the child to the setting. Ultimately, however, the individual was still viewed as different to most people.

Many of the stories of people with disabilities spoken about in the focus groups, particularly pre-intervention, yielded images that portrayed children and adults with disabilities as undesirable or associated with an unpleasant encounter, which affected the extent to which the person was accepted by the community [24–26]. There was some distancing of the person with a disability through the language used to describe individuals. Use of inanimate linguistic objects to describe people with disabilities, e.g. words belonging to the KI-VI class of nouns in the Swahili language such as “kiwete” which has a plural of “viwete” (cripple-cripples), rendered the person as an object. Thus disabilities were viewed as entities, devoid of human life. There was the added tendency to describe a lack, or total absence of ability. This finds resonance in the reification/recognition dichotomy explored by the German philosopher Axel Honneth [50]. He highlighted the human tendency to treat other people as mere objects (reification) instead of responding to them as individuals who are more than just things (recognition). The propensity to abuse vulnerable individuals springs partly from this tendency. However, we also have the capacity to recognize others, which was evident in some of the accounts of persons with disabilities who were known to the respondents, and emphasized in the post-intervention data.

Encountering experts-by-experience who shared their personal narratives, appears to have triggered a reassessment of the meanings associated with disability [32–37]. Confronted with the experiences of people living with disability, the groups developed stronger recognition of the person who has a disability. According to Honneth, this is a prerequisite for empathy [50], and certainly the post-intervention discussions revealed the expression of empathetic views. There was also greater emphasis placed on the agency of persons with disabilities in terms of abilities and facilitating actions by others, and on being human in terms of recognition and inclusion. Allport’s ‘contact hypothesis’ attributes these changes to the deliberate interactions with people living with disabilities [32], which corroborates the findings of Armstrong et al. [34, 35], MacMillan et al. [32], and Schwab [34]. The deliberate exposure of the groups to people with disabilities, who were the frequent subjects of negative images and devaluation, challenged the stereotypical views of persons perceived to be a burden and the ‘objects’ of dehumanizing practice [36–38]. This resonates the findings of other studies that have promoted encounters with people with disabilities in pursuit of attitudinal change [36–39]. Post-intervention, there was a general realization that people with disabilities needed love and respect, just like other human beings. The stories by the experts-by-experience caused many participants to shed tears, and to express new insights into the experiences of people with disabilities, which were commensurate with empathy. The call to act on this new knowledge was shared by many of the groups, who sought to sensitize the community and employ experts-by-experience themselves to affect positive change in their communities.

### Limitations

The sample was comprised of established groups of community health workers and women’s groups. Whilst the value of formally constituted memberships brought mutual familiarity and shared local knowledge to the focus group discussions, gender bias in the women’s groups may be a factor in their discussions, just as the more health focus of the CHWs may have influenced their particular views on disability. Future research could involve other community groups considered to be opinion leaders, such as heads of ten homesteads, village leaders and even traditional healers to give diversity of perceived views. The experts-by-experience originally recruited to deliver the intervention were representative of a range of the most common developmental disabilities (e.g. cerebral palsy, hearing impairment, autism and intellectual disability). However, 2 individuals withdrew their services during the preparation period, which reduced the diversity of personal narratives presented to the groups. This may have affected opportunities to consider the broad spectrum of disability in the groups. Saturation checks on the data for possible follow up in terms of data collection were not made due to financial and time constraints. The questions and prompts used to guide the focus group discussions post-intervention, might have usefully included reflections on the pre-intervention discussions. By asking participants to comment on the views expressed prior to meeting the experts-by-experience, direct capture of any transformations in attitude may have been possible. Whilst the study was able to capture community group responses to disability, and to examine the potentially mediating effect of exposure to experts-by-experience, further research is needed to evaluate intervention potency and effect size.

## Conclusions

The research contributes to the evidence on CBR, demonstrating the potential of a low-cost, relatively brief intervention, which may be applicable in other low-income settings. The very act of employing people with disabilities assigns them a higher status as ‘experts-by-experience’, which is in opposition to the more familiar ‘discounted’ role seen in many communities. The participants wanted the experts to have the same opportunities as others, which extended to being married and having their own home.

The sharing of life experiences through organized community engagement, appears to have facilitated community groups to reflect on, examine and adjust their attitudes towards people with disabilities. The personal narratives served to authenticate the process of learning by the community groups. Listening to and interacting with the experts-by-experience invited the participants to reflect on their own values, against a backdrop of deeply held cultural beliefs and practices. The personal stories guided the participants in viewing the ‘person’ before the ‘disability’. This appeared to be a catalyst for change, both in the attitudes espoused, and in the motivations expressed by the groups. For example, the groups articulated a shared quest to achieve better understanding of disability and to reach more people in their communities. Ultimately, the findings from the current study have implications for mobilizing DPOs, parent or caregiver groups and other rights-based organizations as agents for change in local communities. In this way, a new type of ‘stygmergy’ [28] may be triggered by engaging with the lived experiences of people with disabilities – one that revises the view from the community bringing about recognition of the person - who just happens to have a disability.

## Additional file

Additional file 1: Appendix 1. Pre-intervention guide and Post-intervention guide. (DOCX 14 kb)

## Abbreviations

CBR: Community based rehabilitation
CHW: Community health worker
DPO: Disabled People’s Organization
KEMRI: Kenya Medical Research Institute
UK: United Kingdom
WG: Women group
