# Supplementary material for: Persons with disabilities as experts-by experience: using personal narratives to affect community attitudes in Kilifi, Kenya
Source: BMC Int Health Hum Rights. 2018 May 8;18:18. doi: 10.1186/s12914-018-0158-2 (PMC5941597; doi:10.1186/s12914-018-0158-2)
Supplement: Supplementary file 1 — Appendix 1. Pre-intervention guide and Post-intervention guide. (DOCX 14 kb) [file 12914_2018_158_MOESM1_ESM.docx]

**Additional file 1**

**Pre-intervention Guide**

*English version*

1: Have you had an encounter with a person with a disability? What type of disability? What kind of a person was s/he? What were the difficulties? What could s/he do? What do you think causes disability?

2: Have you had communication problems with a person who has a disability? What were the difficulties? What could s/he do?

3: What can the community do about these disabilities and difficulties experienced by such individuals?

*Giriama version*

1: Udzangwe kuona hedu kushi na mutu mwenye ulemavu? Were anaulemavu wani? Were ahizho? Shidaze were ni noni? Were anadima kuhenda noni? Unafikiri ulemavu unarehewa ni noni?

2: Udzangwe kukala na shida ya kuwasiliana na mutu mwenye ulemavu? Shidaze were ni noni? Were anadima kuhenda noni?

3: Lalo rinadima kuhenda noni dzulu za ulemavu na shida za kuwasiliana ambazo aa atu manazipata?

**Post-intervention Guide**

*English version*

What did you learn from the stories of the expert group?

What else did you learn?

How did the group touch your heart?

What do you think it is important for the community to know?

*Giriama version*

Wadzifundishani kula kwa ngano za kikundi cha atu enye ulemavu?

Wadzifundishani noni kaheri?

Cho kikundi chagutadze moyoo?

Unafikiri ni kitu chani muhimu jamii ni imaye?
